# Two cases of gastrointestinal perforation after radiotherapy in patients receiving tyrosine kinase inhibitor for advanced renal cell carcinoma

**DOI:** 10.1186/1477-7819-10-167

**Published:** 2012-08-20

**Authors:** Takaaki Inoue, Hidefumi Kinoshita, Yoshihiro Komai, Takashi Kawabata, Gen Kawa, Yoshiko Uemura, Tadashi Matsuda

**Affiliations:** 1 Department of Urology, Kansai Medical University in Japan, Hirakata Shinmachi 2 tyoume 3-1, Hirakata City, Osaka, 573-1191, Japan

**Keywords:** Tyrosine kinase inhibitor, Gastrointestinal perforation, Radiosensitivity

## Abstract

We report two cases of gastrointestinal perforation (GIP) after radiotherapy in patients receiving tyrosine kinase inhibitor (TKI) for advanced renal cell carcinoma (RCC). Case 1 was a 61-year-old woman with lung metastases after a radical nephrectomy for a right RCC (cT3aN0M0) treated with interferon-alpha (OIF, 5 MIU, three times per week). She developed lytic metastases of the left femur and the left acetabulum. She was treated with palliative radiotherapy to the metastatic portion (3 Gy × 10 fractions), and 400 mg sorafenib twice per day plus continuing interferon alpha. She experienced sudden left lower abdominal pain after four weeks of treatment, and was diagnosed with a perforation of the sigmoid colon with fecal peritonitis. Case 2 was a 48-year-old man with lung, lymph node, and bone metastases after a radical nephrectomy for a right RCC (cT2N0M0), and was treated with 400 mg sorafenib twice per day. He developed lytic bone metastases of the lumbar vertebrae, which was treated with palliative radiotherapy to L2-4 (3 Gy × 10 fractions). He experienced sudden abdominal pain after two months of radiation treatment, and was diagnosed with a perforation of the sigmoid colon with fecal peritonitis. These cases underwent radiotherapy, and therefore this may be related to the radiosensitivity of TKI.

## Background

Recent advances in the understanding of the molecular biology of advanced and metastatic renal cell carcinomas (RCCs) have led to the development of several systemic therapeutic agents that target vascular endothelial growth factor (VEGF), the mammalian target of rapamycin (mTOR) pathways, and these drugs have shown impressive antitumor efficacy. In particular, the tyrosine kinase inhibitor (TKI) sorafenib, which mainly blocks VEGF pathways, is becoming one of the treatment options for cytokine-refractory RCCs, and a first-line therapy for selected RCC patients. And sunitinib is the first-line therapy in advanced metastatic RCCs [1-3]. It is expected that these TKIs could dramatically improve the progression-free survival and overall survival of advanced RCC patients. On the other hand, some adverse effects (AE) that did not occur with cytokine therapy may occur when using TKIs, and may develop into serious and fatal conditions in some cases.

Here, we report two cases of gastrointestinal perforation (GIP) after radiotherapy in patients receiving TKI for advanced RCC.

## Case presentation

### Patient 1

**Figure 1:**
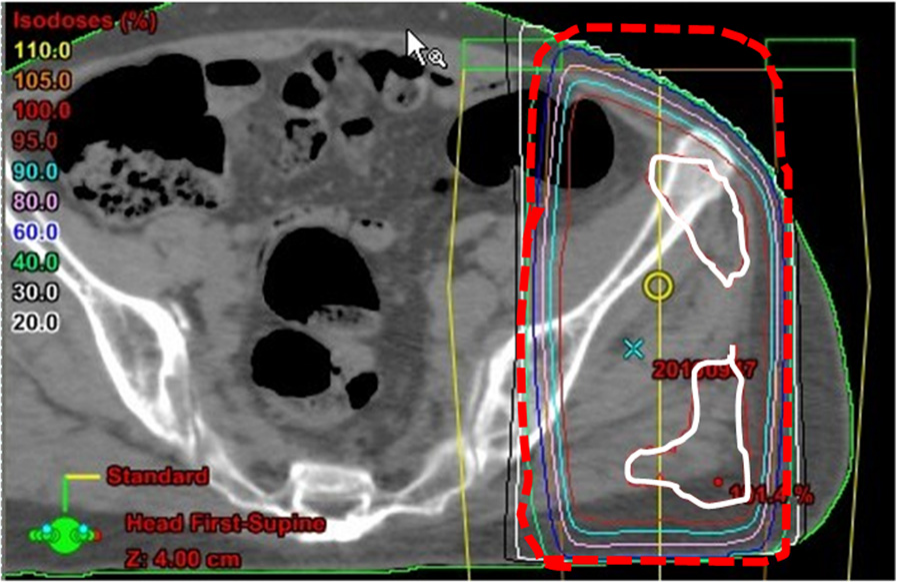
Computed tomography revealed the irradiated area; the white area received 100%, and the dotted-area received 60% of the irradiation.

**Figure 2:**
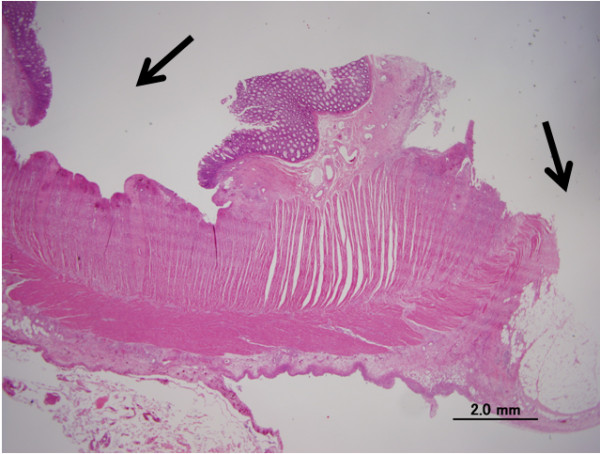
The arrows point at the ulcer and the perforated portion in which a necrotic exudate was observed on the face of the membrane serosa.

**Figure 3:**
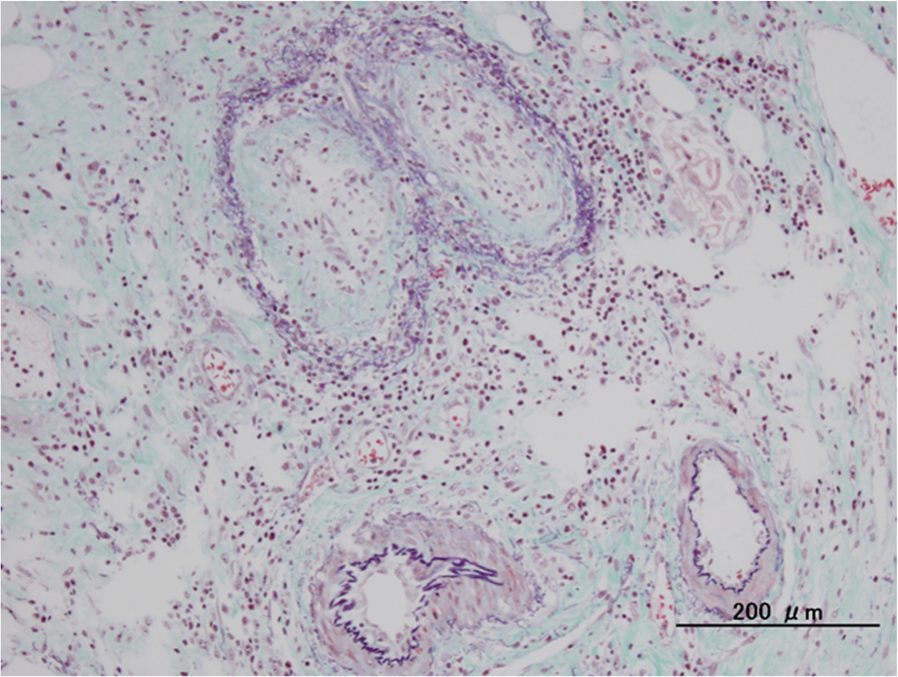
An Elastica van Gieson stain revealed that there were narrowed blood vessels with some thrombus formation and organization in the vascular lumen.

A 61-year-old woman received a radical nephrectomy for a right RCC (cT3aN0M0) in April 2009. The pathological findings confirmed a RCC, clear cell carcinoma, pT2, G2. She developed multiple lung metastases in January 2010. She was classified into the poor risk category of the Memorial Sloan-Kettering Cancer Center risk classification (MSKCC-risk), and was treated with interferon alpha (Sumiferon, 6 MIU, three times per week). However, the lung metastases gradually increased, and she presented with dysbasia and left lower limb pain. On examination, she had new metastatic lesions of the left femur and left acetabulum, which were treated with palliative radiotherapy (3 Gy x 10 fractions) (Figure 1). She was started on sorafenib, 400 mg twice per day, plus continuing interferon alpha seven days after beginning radiotherapy. After four weeks of treatment, she suffered from sudden left lower abdominal pain and abdominal guarding, and on that day, an examination revealed signs of peritonitis. She received an emergent laparotomy. A perforation of the sigmoid colon was observed during the operation, and a sigmoidectomy and colostomy were performed. Around the perforation in the sigmoid colon, two ulcers were observed, and the perforation was solitary. No tumors or diverticulitis were observed. A pathological exam revealed that there was a remarkable, full-thickness invasion of eosinophilic leukocytes around the ulcer, and also invasion by neutrophilic leukocytes. There was a necrotic exudate on the membrane serosa of the perforation, and narrow blood vessels with some thrombus formation and organization in the vascular lumen around the circumference of the perforation were observed (Figures 2 and 3). However, there were no specific findings except for this serositis and narrowed blood vessels. After the operation, she did not recover, and died on postoperative day 29 due to severe sepsis, and multiple organ dysfunction.

### Patient 2

A 48-year-old man received a radical nephrectomy for a right RCC (cT2N0M0) in February 2005. The pathological findings confirmed a RCC, clear cell carcinoma, pT2, G1. He developed some new lung metastases in March 2006 (MSKCC-risk: intermediate). Although he was started on interferon alpha (Sumiferon, 6 MIU, three times per week), the lung metastases gradually increased. Furthermore, lesions appeared in the right iliac bone, thoracic vertebrae (Th3) and mediastinal lymph nodes. He was started on sorafenib 400 mg twice per day in July 2008, and was treated with palliative radiotherapy to the right iliac bone and Th3 (3 Gy x 10 fractions). In August 2009, he developed three new brain metastatic lesions 15 mm in diameter, which were treated by cyberknife. However, we continued the sorafenib at his request. He then developed left lower paralysis and weakness in October 2009. Furthermore, an MRI of the lumbar spine showed lytic bone metastases in L2-4, which were treated with palliative radiotherapy (3 Gy x 10 fractions). Two months after radiotherapy, he presented with sudden abdominal pain, distention, and guarding. On that day, an examination revealed signs of peritonitis. He received an emergent laparotomy. A perforation of the sigmoid colon was observed during the operation. A sigmoidectomy and colostomy were performed. The perforation was solitary around the perforation in the sigmoid colon. No tumors or diverticulitis were observed on the circumference of the colon. We regret that we did not submit a tissue sample of the perforation in the sigmoid colon for pathological exam. After the operation, he recovered and was discharged from hospital. Unfortunately, he died three months later due to cancer progression.

## Discussion

The gastrointestinal complications of patients receiving tyrosine kinase inhibitor consist of nausea, vomiting (40 to 53%) and diarrhea (43 to 53%), all of which are relatively common, as well as gastrointestinal bleeding (2.4%) and gastrointestinal perforation (0 to 0.2%), which are relatively rare. However, these complications do pose a material risk because in some cases, they may be quite serious or even fatal [1,2,4].

The incidence rate of GIP during TKI therapy is generally rare**.** In a pivotal study on sorafenib for the treatment of advanced RCCs, 451 patients with RCCs received sorafenib with no reports of GIP [1], but two (0.2%) instances of GIP occurred in 544 patients with advanced RCC, and in 257 patients with gastrointestinal stromal tumors receiving sunitinib [2,4]. There was a 0.7% incidence of GIPs during treatment for unresectable/metastatic renal cell carcinomas according to the third interim analysis report of the postmarketing Surveillance Review of Sorafenib in Japan. However the details have not been reported [5]. One (0.3%) case of GIP was reported in the randomized phase Pazopanib trial [6] and another (0.2%) GIP case was also reported in the phase III Bevacizumab trial [7] for locally advanced or metastatic RCC. However, in the phase III Axitinib trial [8] for advanced RCC, GIP was not reported. In the phase II Axitinib trial [9] for metastatic melanoma, one (3.1%) case of GIP was reported. However, in the use of mTOR inhibitors such as Everolimus, and Temsirolimus for advanced RCC, GIPs were not reported. [10,11]. Recently, one (3.8%) case of GIP was reported in the phase 1 evaluation of telatinib, a VEGF receptor tyrosine kinase inhibitor, in combination with bevacizumab in subjects with advanced solid tumors [12]. On the other hand, some recent case reports of GIP with sunitinib or sorafenib for RCCs have been published [13-15]; these studies are in Table 1. Except for one patient that was diagnosed with a metastatic cancer at the perforation site, the remaining six patients did not have malignant findings, which may mean that colonic tumor involvement and cancer metastases are not the cause of the GIPs during TKI for RCC in many cases. 

**Table 1:** Summarized data of patients with GIP during TKI treatment for RCC

**No.**	**Age**	**Type of tumor**	**Metastatic site**	**Previous treatment**	**Present treatment**	**With palliative adiation (Gy)**	**Month after TKI (mo)**	**Initial symptom**	**Perforation location**	**Treatment**	**Pathological findings in perforation**	**Follow up**	**Author**
1.	50	RCC	Lung	Inferon	Sunitinib (50)	(−)	6	Abdominal pain	Ascending colon	Surgery	No malignancy	Alive	Hoshino *et al*.
2.	60	RCC	Right Pelvis bone	Inferon	Sunitinib (50)	(−)	0	Abdominal pain	Jejunum	Surgery	Cancer metastases	Death after 3- mo	Hoshino *et al.*
3.	Unknown	RCC	Lung	High dose Interleukin- 2	Sunitinib (50)	(−)	13	Abdominal pain	Ascending	Surgery	No malignancy	Unknown	Flaig *et al*.
4.	Unknown	RCC	Lung	High-dose Interleukin-2	Sunitinib (37.5)	(−)	20	Abdominal pain	Ascending colon	Conservative	Unknown	Unknown	Flaig *et al*.
5.	61	RCC	Skin Lung spine	(−)	Sunitinib (800)	L3-5 (8)	2	Abdominal pain	Transverse colon Sigmoid colon	Conservative	No malignancy	Death after 1- day	Peters
6.	48	RCC	Lung Lymph node Multiple bone	Interferon	Sorafenib (800)	Th3, L2-4 (30)	14	Abdominal pain	Sigmoid colon	Surgery	No malignancy	Death After3- mo	Our case
7.	61	RCC	Lung Left femoral bone	Interferon	Sorafenib (800)	Left femoral (30)	1	Abdominal pain	Sigmoid colon	Surgery	No malignancy	Death after 29 days	Our case

GIP, gastrointestinal perforation; RCC, renal cell carcinoma; TKI, tyrosine kinase inhibitor.

The radiotherapy itself does not generally have many adverse effects. Eifel *et al*. reported that the incidence of sigmoid perforation caused by high-dose radiotherapy for cervical cancer was 0.6% at 20 years, with most perforations occurring less than 30 months (3 to 98 months) after treatment, but the fatality rate was 41% [16]. The tolerance dose of radiation for the normal intestine is 50 to 60 Gy [17,18]. In the present study, our cases with GIP were irradiated as palliative therapy (3 Gy x 10 fractions) against bone metastases, and did not reach the tolerance dose. Patient 1 started sorafenib at seven days after beginning radiotherapy, and the bowel perforation occurred after five weeks of radiotherapy. Patient 2 received palliative radiotherapy to L2-4 at fifteen months after beginning sorafenib, and the bowel perforation occurred after two months of radiotherapy. Thus, the GIPs in our cases occurred one to two months after the cessation of radiotherapy. We suspect that our cases may be affected by the TKIs, because our GIPs occurred earlier after the cessation of radiotherapy, and in spite of the low dose of radiation, which does not reach the tolerance dose for the intestine. Recently, several preclinical studies showed that some anti-angiogenic compounds, including TKI, may have a radiosensitizing effect by inhibiting the VEGF pathway in normal and cancer cells [19,20]. Normal cells, including endothelial cells, would also increase their radiosensitivity according to the action of the anti-angiogenic therapy, and seem to be more susceptible to damage from radiotherapy, although the mechanism is unclear [21]. We hypothesized the cause of the GIPs in our cases might be related to the radiosensitive nature of TKIs. However, it is still unproven whether TKIs truly have potential radiosensitive activity.

The pathological findings around the perforation may be important. Peters *et al*. reported that there were some blood vessels with thrombus formation on the perforation spot [14]. In our cases, there was also serositis of the perforation and narrowed blood vessels with some thrombus formation and organization in their vascular lumen around the circumference of the perforation. However, it is unclear whether these pathological findings are specific for GIP induced by TKI.

## Conclusion

Here, we experienced two cases of GIP after radiotherapy in patients receiving TKI for advanced RCCs. Although GIPs during TKI therapy for RCC are rare, a serious or fatal outcome may be expected. In particular, when radiotherapy is performed with TKI, the radiosensitizing effect of the anti-angiogenic therapy may increase the risk of GIP. To clarify the risk of GIP, the accumulation of more data on when radiotherapy is performed with TKI therapy is required.

## Consent

Written informed consent was obtained from the next of kin of the patient for the publication of this case report and the accompanying images. A copy of the written consent is available for review by the Editor-in-Chief of this medical journal.

## Abbreviations

AE: Adverse effect; GIP: Gastrointestinal perforation; MRI: Magnetic resonance imaging; mTOR: Mammalian target of rapamycin; RCC: Renal cell carcinoma; Th: Thoracic vertebrae; TKI: Tyrosine kinase inhibitor; VEGF: Vascular endothelial growth factor.

## Competing interests

The authors declare that they have no competing interests.

## Authors’ contributions

All authors read and approved the final manuscript.
